# Structural Identifiability of Dynamic Systems Biology Models

**DOI:** 10.1371/journal.pcbi.1005153

**Published:** 2016-10-28

**Authors:** Alejandro F. Villaverde, Antonio Barreiro, Antonis Papachristodoulou

**Affiliations:** 1 Department of Engineering Science, University of Oxford, Oxford, United Kingdom; 2 Department of Systems & Control Engineering, University of Vigo, Vigo, Spain; University of Tokyo, JAPAN

## Abstract

A powerful way of gaining insight into biological systems is by creating a nonlinear differential equation model, which usually contains many unknown parameters. Such a model is called structurally identifiable if it is possible to determine the values of its parameters from measurements of the model outputs. Structural identifiability is a prerequisite for parameter estimation, and should be assessed before exploiting a model. However, this analysis is seldom performed due to the high computational cost involved in the necessary symbolic calculations, which quickly becomes prohibitive as the problem size increases. In this paper we show how to analyse the structural identifiability of a very general class of nonlinear models by extending methods originally developed for studying observability. We present results about models whose identifiability had not been previously determined, report unidentifiabilities that had not been found before, and show how to modify those unidentifiable models to make them identifiable. This method helps prevent problems caused by lack of identifiability analysis, which can compromise the success of tasks such as experiment design, parameter estimation, and model-based optimization. The procedure is called STRIKE-GOLDD (STRuctural Identifiability taKen as Extended-Generalized Observability with Lie Derivatives and Decomposition), and it is implemented in a MATLAB toolbox which is available as open source software. The broad applicability of this approach facilitates the analysis of the increasingly complex models used in systems biology and other areas.

## Author Summary

Advances in computing power have facilitated the development of increasingly larger dynamic models of biological processes, which usually have many unknown parameters. Often times, such models contain parameters that are structurally unidentifiable, i.e., they cannot be uniquely determined from experiments. Any parameter estimation algorithm will fail when trying to estimate unidentifiable parameters, leading to waste of resources and possibly wrong model predictions. Hence, it is essential to assess structural identifiability before exploiting a model. However, performing such analysis can be hard, especially as models become increasingly complicated. To address this challenge, we developed a methodology for structural identifiability analysis that aims at generality—it can handle any analytic model written as a set of ordinary differential equations—and computational efficiency—it includes features that facilitate the analysis of large systems. We provide an implementation of the methodology as a MATLAB toolbox called STRIKE-GOLDD. We illustrate its applicability to systems biology models of genetic, signalling, metabolic, and pharmacokinetic networks, showing which of them are unidentifiable and how they can be made identifiable.

This is a *PLOS Computational Biology* Methods paper

## Introduction

Mathematical modelling has become a fundamental tool in present day biology [1], and system identification is one of the key tasks of this process [2]. Building a dynamic model usually requires establishing the values of some unknown parameters, which raises the issue of parameter identifiability [3]. A model is structurally identifiable if it is possible to determine the values of its parameters from observations of its outputs and knowledge of its dynamic equations [4]. While the related concept of *practical* identifiability refers to quantifying the uncertainty in parameter values when estimated from noisy measurements, *structural* identifiability does not take into account limitations caused by the quality or availability of experimental data. It is, however, a necessary (*a priori*) condition for practical identifiability, which, in turn, is a prerequisite for model calibration, also known as parameter estimation [5]. Any identification efforts aimed at estimating unidentifiable parameters will fail, leading to wrong estimates, waste of resources, and possibly misleading model predictions [6]. Furthermore, if structural unidentifiability is mistaken for practical unidentifiability, it may lead to trying to overcome it by investing additional efforts in designing and performing new experiments [7], which will nevertheless be sterile. Hence it is essential to assess the structural identifiability of any unknown parameters in a model before attempting to calibrate it. As stressed in the conclusions of a recent parameter estimation challenge [8], “modelers must avoid creating structurally unidentifiable parameters that can never be estimated”. However, in real applications structural identifiability is seldom checked before performing parameter estimation [9]. This is at least partly due to the computational complexity of the problem: structural identifiability methods generally require symbolic manipulations, which can quickly give rise to long expressions as the system size increases [10].

This is a major challenge in systems biology, as the models constructed are increasingly complex, large [11], and more difficult to identify [8]. However, the development of structural identifiability tools has been lagging behind, and, despite the wide variety of methods developed for this task (some of which have publicly available implementations [12–16]), the analysis of some models remains elusive. For example, although recent improvements in efficiency [16, 17] have enabled the analysis of increasingly large rational models (those that can be expressed as fractions of polynomial functions), non-rational systems such as those including trigonometric expressions or Hill-type kinetics (which are common in mechanical and biochemical models, respectively) can currently be analysed only for small sizes. While in certain cases non-rational models can be rewritten in rational form, by introducing additional variables and equations, it is not always possible or convenient to do so. Furthermore, the results obtained for the rational counterpart are not necessarily valid for the original non-rational model in the case of unidentifiability [18]. Recent studies [9, 10, 19–21] show that, in general, the choice of a structural identifiability method involves trade-offs between generality of the application, computational cost, and level of detail of the results. In conclusion, there is currently a lack of structural identifiability methods of the sufficient generality and robustness to be applied to nonlinear models of general form and realistic size [21, 22].

To address this issue, we propose a methodology applicable in principle to any analytic system and geared towards computational efficiency. This method approaches local structural identifiability as a generalized version of observability, a classic concept in systems and control theory [23]. A system is observable if it is possible to determine its internal state from output measurements in finite time. If the model parameters are considered as state variables with zero dynamics, structural identifiability analysis can be recast as a generalization of observability analysis [17, 24, 25]. In this way it is possible to assess the structural identifiability of nonlinear systems using results from differential geometry [21]. Essentially, identifiability is determined by calculating the rank of a generalized observability-identifiability matrix, which is constructed using Lie derivatives. When this rank test classifies a model as unidentifiable, the procedure determines the subset of identifiable parameters. In some cases it is also possible to find identifiable combinations of the remaining parameters. This approach is directly applicable to many models of small and medium size; larger systems can be analysed using additional features of the method. One of them is decomposition into more tractable submodels, which is performed with a combinatorial optimization metaheuristic as in [26]. Another possibility is to build identifiability matrices with a reduced number of Lie derivatives. In some cases these additional procedures allow to determine the identifiability of every parameter in the model (complete case analysis); when such result cannot be achieved, at least partial results—i.e. identifiability of a subset of parameters—can be obtained.

We illustrate the applicability of this method to systems biology models of different types, including genetic, signalling, metabolic, and pharmacokinetic networks. Some of them are non-rational systems exhibiting Hill kinetics, that is, with expressions containing terms of the form *k*_1_*x*^*n*^/(*k*_2_ + *x*^*n*^), such as the Goodwin model of transcriptional repression [27], the mitogen-activated protein kinase (MAPK) signalling cascade [28], and the genetic network that controls the circadian clock in *Arabidopsis thaliana* [29]. Other models analysed here include drug uptake into hepatocytes [19], NF-*κ*B [30] and JAK/STAT [31] signalling pathways, and the central carbon metabolism of Chinese hamster ovary cells [32]. These case studies include models whose identifiability had not been previously determined, and for some of them we found unidentifiabilities that had not been reported before. In those cases, we obtained identifiable reparameterizations by removing redundant parameters and fixing the values of other parameters *a priori*.

## Methods

We consider dynamic models described by ordinary differential equations of the following general form:

$$M:\left\{\begin{array}{ccc} \dot{x}\left(t\right) & = & f[x(t),u(t),p],\\ y(t) & = & g[x(t),p],\\ x_{0} & = & x(t_{0},p) \end{array}\right.$$ (1)

where *f* and *g* are analytic (and therefore infinitely differentiable) vector functions, $p\in R^{q}$ is a real-valued vector of parameters, $u\left(t\right)\in R^{r}$ is the input vector, $x\left(t\right)\in R^{n}$ the state variable vector, and $y\left(t\right)\in R^{m}$ the measurable output, also called the observables vector. In Eq (1) the dependence on the parameters *p* is made explicit, but it will be usually dropped for ease of notation. Parameter *p*_*i*_ is structurally globally identifiable (s.g.i.) if it can be uniquely determined from the system output, that is, if for almost any $p^{*}\in R^{q}$ (i.e., for any *p* except those belonging to a set of measure zero) the following property holds [5, 33]:

$$y\left(t,\hat{p}\right)=y\left(t,p^{*}\right)\Rightarrow \hat{p_{i}}=p_{i}^{*}$$ (2)

A parameter *p*_*i*_ is structurally locally identifiable (s.l.i.) if for almost any p* there is a neighbourhood *V*(p*) in which Eq (2) holds. A model *M* is said to be s.g.i. if all its parameters are s.g.i., and s.l.i. if all its parameters are s.l.i. If Eq (2) does not hold in any neighbourhood of p*, parameter *p*_*i*_ is structurally unidentifiable (s.u.), and a model *M* is s.u. if at least one of its parameters is s.u.

### Observability of nonlinear systems

In this work we consider identifiability as an augmented observability property. We begin the description of the approach by defining observability and showing how it can be assessed. A system is (locally) observable at a state *x*_0_ if there exists a neighbourhood *N* of *x*_0_ such that every other state *x*_1_ ∈ *N* is distinguishable from *x*_0_. Two states *x*_0_ ≠ *x*_1_ are said to be distinguishable when there exists some input *u*(*t*) such that *y*(*t*, *x*_0_, *u*(*t*)) ≠ *y*(*t*, *x*_1_, *u*(*t*)), where *y*(*t*, *x*_*i*_, *u*(*t*)) denotes the output function of the system for the input *u*(*t*) and initial state *x*_*i*_(*i* = 0, 1).

The concept of observability was initially formulated by Kalman for linear systems [34], and then extended to the nonlinear case by Hermann and Krener [23]. For a nonlinear system given by Eq (1) it is possible to obtain information about the states *x* from its outputs *y* by calculating the derivatives $\dot{y},\ddot{y},\ldots$. These differentiations are performed by taking Lie derivatives of the output function *g*. The Lie derivative of *g* with respect to *f* is:

$$L_{f}g\left(x\right)=\frac{\partial g(x)}{\partial x}f\left(x,u\right)$$ (3)

For a system with *n* states and *m* outputs, $\frac{\partial }{\partial x}g\left(x\right)$ is an *m* × *n* matrix, and $L_{f}g\left(x\right)=\frac{\partial g(x)}{\partial x}f\left(x,u\right)$ is an *m* × 1 column vector. The *i*^*th*^ order Lie derivatives are recursively defined as follows:

$$\begin{array}{ccc} L_{f}^{2}g\left(x\right) & = & \frac{\partial L_{f}g\left(x\right)}{\partial x}f\left(x,u\right)\\ & \cdots & \\ L_{f}^{i}g\left(x\right) & = & \frac{\partial L_{f}^{i-1}g\left(x\right)}{\partial x}f\left(x,u\right) \end{array}$$ (4)

Stacking *n* sub-matrices, we obtain the nonlinear observability matrix:

$$O\left(x\right)=\left(\begin{array}{c} \frac{\partial }{\partial x}g\left(x\right)\\ \frac{\partial }{\partial x}\left(L_{f}g\left(x\right)\right)\\ \frac{\partial }{\partial x}\left(L_{f}^{2}g\left(x\right)\right)\\ \vdots \\ \frac{\partial }{\partial x}\left(L_{f}^{n-1}g\left(x\right)\right) \end{array}\right)$$ (5)

We can now formulate the *Observability Rank Condition (ORC)* as follows: if the system given by Eq (1) satisfies $\text{rank}(O\left(x_{0}\right))=n$, where O is defined by Eq (5), then it is (locally) observable around *x*_0_ [35].

The rank condition provides a result about *local* observability of *any* possible state *x*_0_. That is, if the matrix is full rank then for every state *x*_0_ there exists a neighbourhood *N*(*x*_0_) in which *x*_0_ can be distinguished from any other state x*. In other words, every state can be distinguished from its neighbours, but not necessarily from other distant states. In contrast, *global* observability is a property that must hold for every possible *N*(*x*_0_). The difference is clearly shown with the following example [23]:

$$\dot{x}=u,\;\;\;y_{1}=\cos\left(x\right),\;\;\;y_{2}=\sin\left(x\right)$$ (6)

While this system satisfies the observability rank condition and is therefore locally observable, it is not globally observable because it is impossible to distinguish between *x*^0^ and *x*^*k*^ = *x*^0^ + 2*kπ*, for any integer *k*.

We remark that the observability rank condition does not require the assumption of constant inputs *u*; analytic differentiable input functions can be used [36, 37]. As noted in [38], this entails that *u* can be treated symbolically in rank calculations.

### Structural identifiability as augmented observability: The OIC

While identifiability problems can be addressed by a number of techniques not explicitly related to nonlinear observability, it is possible to consider the parameters *p* as additional states with trivial dynamics $\dot{p}=0$ and, in this way, the identifiability problem can be recast in the framework of observability [17, 21, 24]. Thus, by augmenting the state variable vector so as to include model parameters, $\tilde{x}=[x,p]$, we obtain a generalized observability-identifiability matrix, $O_{I}\left(\tilde{x}\right)$:

$$O_{I}\left(\tilde{x}\right)=\left(\begin{array}{c} \frac{\partial }{\partial \tilde{x}}g\left(\tilde{x}\right)\\ \frac{\partial }{\partial \tilde{x}}\left(L_{f}g\left(\tilde{x}\right)\right)\\ \frac{\partial }{\partial \tilde{x}}\left(L_{f}^{2}g\left(\tilde{x}\right)\right)\\ \vdots \\ \frac{\partial }{\partial \tilde{x}}\left(L_{f}^{n+q-1}g\left(\tilde{x}\right)\right) \end{array}\right)$$ (7)

With this formulation we can define a generalized *Observability-Identifiability Condition (OIC)* as follows: if the system given by Eq (1) satisfies $\text{rank}(O_{I}\left({\tilde{x}}_{0}\right))=n+q$, it is (locally) observable and identifiable in a neighbourhood $N({\tilde{x}}_{0})$ of ${\tilde{x}}_{0}$.

Since we have recast the analysis of structural identifiability as a particular case of observability, the same remark that was made in the preceding subsection about the difference between local and global properties applies here.

It has been noted [39] that in certain cases a system may become unreachable for specific values of the initial conditions, leading to the impossibility of determining the values of parameters classified as identifiable by structural identifiability methods. This situation can be detected if $\text{rank}(O_{I}\left({\tilde{x}}_{0}\right))$ is calculated using a vector of specific initial conditions instead of a generic symbolic vector.

Finally, we note that the idea of treating parameters and state variables similarly is also adopted by estimation methods such as extended Kalman filtering [40]. However, the context is different, since the goal of such techniques is to determine the value of states and parameters from data, while structural identifiability analysis aims at establishing whether such estimation is theoretically possible.

### Assessing the OIC efficiently

In practice, checking the aforementioned Observability-Identifiability Condition (OIC) is often computationally inefficient (or even infeasible) because building $O_{I}$ and calculating its rank is a highly demanding, memory-consuming task. Fortunately, sometimes this cost can be decreased by building a smaller matrix. Let us first note that each of the *n* + *q* sub-matrices vertically stacked in the generalized observability-identifiability matrix of Eq (7) has dimension *m* × (*n* + *q*), and the full matrix $O_{I}$ has dimensions (*m* ⋅ (*n* + *q*)) × (*n* + *q*). Therefore it may not be necessary to calculate the *n* + *q* − 1 Lie derivatives in order to test whether $O_{I}$ is full rank, since full rank may be achieved with a lower number of derivatives. The minimum number of Lie derivatives for which the matrix may be full rank is

$$n_{d}=\left\lceil \frac{n+q}{m}-1\right\rceil$$ (8)

that is, the smallest integer not less than (*n* + *q*)/*m* − 1, where *n*, *q*, and *m* are the numbers of states, parameters, and outputs, respectively. The maximum number of Lie derivatives is also known *a priori*: derivatives of order higher than *n* + *q* − 1 cannot increase the matrix rank [38]. Having lower and upper bounds for the necessary Lie derivatives is an advantage of this methodology compared to, e.g., power series approaches, for which the maximum number of derivatives is in principle infinite [10].

Our method builds $O_{I}$ recursively. Once *n*_*d*_ is reached, addition of a new Lie derivative is followed by calculation of the rank. This process is repeated until the maximum number *n* + *q* − 1 is reached, or until adding a new Lie derivative does not increase the matrix rank; in both cases no further derivatives are necessary [38]. At that point, if $O_{I}$ is full rank the corresponding model is observable and identifiable, as seen in the previous subsection. If $O_{I}$ is not full rank, the algorithm proceeds to find identifiable parameters, as explained in the following subsection.

Further improvements in the computational burden can be obtained by calculating the rank numerically instead of symbolically. A way in which this can be performed is by replacing the symbolic variables in the $O_{I}$ with prime numbers to minimize the risk of accidental cancellations, which would reduce the rank.

### Determining identifiability of individual parameters

If $O_{I}$ is not full rank, the Observability-Identifiability Condition (OIC) does not inform us about which parameters are identifiable and which are not. This can be achieved by realizing that each column of $O_{I}$ corresponds to a parameter-to-output relation (or state-to-output):

$$\left(\begin{array}{cccc} \frac{\partial }{\partial x_{1}}g\left(\tilde{x}\right) & \frac{\partial }{\partial x_{2}}g\left(\tilde{x}\right) & \cdots & \frac{\partial }{\partial p_{q}}g\left(\tilde{x}\right)\\ \frac{\partial }{\partial x_{1}}\left(L_{f}g\left(\tilde{x}\right)\right) & \frac{\partial }{\partial x_{2}}\left(L_{f}g\left(\tilde{x}\right)\right) & \cdots & \frac{\partial }{\partial p_{q}}\left(L_{f}g\left(\tilde{x}\right)\right)\\ \vdots & \vdots & \vdots & \vdots \\ \frac{\partial }{\partial x_{1}}\left(L_{f}^{n+q-1}g\left(\tilde{x}\right)\right) & \frac{\partial }{\partial x_{2}}\left(L_{f}^{n+q-1}g\left(\tilde{x}\right)\right) & \cdots & \frac{\partial }{\partial p_{q}}\left(L_{f}^{n+q-1}g\left(\tilde{x}\right)\right) \end{array}\right)$$

Therefore, if deleting the *i*^*th*^ column of the generalized observability-identifiability matrix does not change its rank, then the corresponding *i*^*th*^ state (parameter) is non-observable (unidentifiable). This fact can be exploited to determine which of the parameters in an unidentifiable model are identifiable and which are not, using a sequential procedure: after the matrix rank has been calculated and the model has been found to be unidentifiable, each of the columns in $O_{I}$ corresponding to a particular parameter is removed one by one and the rank is recalculated. In this way the identifiability of each of the parameters is evaluated.

### Finding identifiable combinations of otherwise unidentifiable parameters

The procedure outlined in the preceding subsections classifies the model parameters as either identifiable of unidentifiable. A question that naturally follows is: are there combinations of the unidentifiable parameters which are themselves identifiable? If the answer is affirmative, the model can be reparameterized and converted to a structurally identifiable model. However, this is a difficult problem, which few methods can address, and only for models of moderate size. An example is COMBOS [41, 42], which is based on differential algebra. Here we suggest an approach based on ideas presented in [43, 44] and on the method for finding symmetries proposed by [38]; related work has been recently presented in [45].

The procedure is as follows: if $O_{I}$ is rank-deficient, remove the columns corresponding to identifiable parameters and obtain a reduced submatrix, $O_{U}$ [38]. Then, obtain a basis for the kernel (null space) of this matrix, $N(O_{U})$ (step 2 in [44]). Its coefficients define one or several partial differential equations whose solution(s) are the identifiable combinations (step 3 in [44]). This procedure is illustrated in the Methods section with the JAK/STAT signalling pathway, for which an identifiable combination of two parameters is found. While this example shows the potential of this procedure, it must be acknowledged that the computational complexity of calculating the kernel of $O_{U}$ limits its applicability to models with a moderate number of unidentifiable parameters.

### Decomposing large models into submodels to facilitate their analysis

The methodology described in the previous subsections can be used to analyse the identifiability of whole models and, if the model is unidentifiable, of its parameters individually. However, since it relies heavily on symbolic operations, it may be computationally infeasible for large or complex models. It should be noted that the main limiting operations are:

- Obtaining high order Lie derivatives to build $O_{I}\left(\tilde{x}\right)$.
- Calculating the rank of the resulting $O_{I}\left(\tilde{x}\right)$.

The minimum number of derivatives necessary for building $O_{I}\left(\tilde{x}\right)$ is given by *n*_*d*_ as defined in Eq (8). The limit of what is computationally possible is difficult to quantify *a priori*, since it depends on the model equations and the machine used in the calculations. As a rule of thumb, analyses involving *n*_*d*_ ≥ 10 are infeasible except for very small models. As model size or complexity increases, this upper bound decreases; some examples will be shown in the Results section.

One solution is to decompose those models into smaller submodels whose analysis is possible computationally. Thus, we seek to decompose a model *M* into submodels {*M*_1_, *M*_2_, …} which require few Lie derivatives for their analysis, that is, they have a small *n*_*d*_. Each submodel *M*_*sub*_ includes a subset of the model states, *x*_*sub*_. Its outputs, *y*_*sub*_, are the outputs of *M* which are functions of at least one state included in *x*_*sub*_. The submodel parameters and inputs are those appearing in the equations of *x*_*sub*_ and *y*_*sub*_. There may be states that appear in the equations of *x*_*sub*_ or *y*_*sub*_ but are not part of *x*_*sub*_; they are considered as additional unknown parameters of *M*_*sub*_.

The submodels can be found by optimization as follows. For each submodel *M*_*i*_ we select a subset of the states in *M* by performing a combinatorial optimization where we minimize *n*_*d*_:

$$\underset{s}{\text{min}}\;n_{d}\left(s\right)$$ (9)

where **s** = {*s*_1_, *s*_2_, …, *s*_*n*_} is a binary vector of size *n*, whose entries *s*_*j*_ ∈ {0, 1} denote inclusion (*s*_*j*_ = 1) or exclusion (*s*_*j*_ = 0) of the corresponding state. The combinatorial optimization is performed with the Variable Neighbourhood Search metaheuristic [46]. We carry out *n* optimizations (one per state); in the *j*^*th*^ optimization we force *s*_*j*_ = 1, so that each state appears in at least one solution. This, in turn, guarantees that all the parameters will eventually be evaluated. A penalty term is included in the objective function to penalize solutions that have more states than a chosen maximum.

Apart from this optimization-based decomposition, it may sometimes be useful to specify a particular submodel in order to explore the identifiability of a specific part of the model.

### Assessing identifiability of decomposed models

Let us clarify how we can conclude identifiability of a parameter from analysis of a submodel. As an example, consider *M* to be the model of *Arabidopsis thaliana* described in the Results section; its equations are given in the Supplementary Information (S1 Text). Let us consider a submodel *M*_*sub*_ consisting of two states, *x*_*sub*_ = {*x*_1_, *x*_7_}. The equations of *M*_*sub*_ are those that correspond to the states {*x*_1_, *x*_7_}, that is:

$$\{\begin{array}{c} {\dot{x}}_{1}=n_{1}\frac{x_{6}^{a}}{g_{1}^{a}+x_{6}^{a}}-m_{1}\frac{x_{1}}{k_{1}+x_{1}}+q_{1}x_{7}u\left(t\right),\\ {\dot{x}}_{7}=p_{3}-m_{7}\frac{x_{7}}{k_{7}+x_{7}}-\left(p_{3}+q_{2}x_{7}\right)u\left(t\right),\\ x_{1}\left(0\right)=0,x_{7}\left(0\right)=0 \end{array}$$ (10)

The outputs of *M*_*sub*_ are those outputs of *M* which are functions of at least one of the states in *M*_*sub*_ (in this example, *y*_1_ = *x*_1_). The parameters and inputs of *M*_*sub*_ are those present in Eq (10): respectively, {*n*_1_, *g*_1_, *a*, *m*_1_, *k*_1_, *q*_1_, *p*_3_, *m*_7_, *k*_7_, *q*_2_} and *u*. Additionally, we must also include as parameters the states that do not belong to *x*_*sub*_ but appear in Eq (10) or in *y*_*sub*_ (in this case, *x*_6_). Thus in this example the submodel parameters would be {*n*_1_, *g*_1_, *a*, *m*_1_, *k*_1_, *q*_1_, *p*_3_, *m*_7_, *k*_7_, *q*_2_, *x*_6_}. By including states such as *x*_6_ as parameters we are considering them as unknown and constant. In contrast, if they were included as inputs to the submodel, we would be implicitly assuming that they provide sufficient excitation for identification purposes. Thus, including them as parameters is a conservative assumption in terms of identifiability. Therefore, if a parameter is classified as identifiable in a submodel under these conditions, it will also be identifiable when considering the whole model.

### Building $O_{I}$ with less than *n*_*d*_ Lie derivatives

When the *n*_*d*_ of the full model is so high that it is not feasible to build $O_{I}$, one solution is to decompose the model into smaller submodels as described in the previous subsections. Another possibility is to build $O_{I}$ with *i* < *n*_*d*_ derivatives. In this case we know that full rank cannot be achieved, so even if the model is identifiable we will not be able to determine it in this way. However, it may be possible to determine identifiability of at least *some* of the parameters. Note that this procedure can be helpful exactly in the same circumstances as decomposition. In some cases one approach will be more appropriate than the other, but both can be used to determine the identifiability of different parameters, and may therefore be complementary.

**Fig 1:**
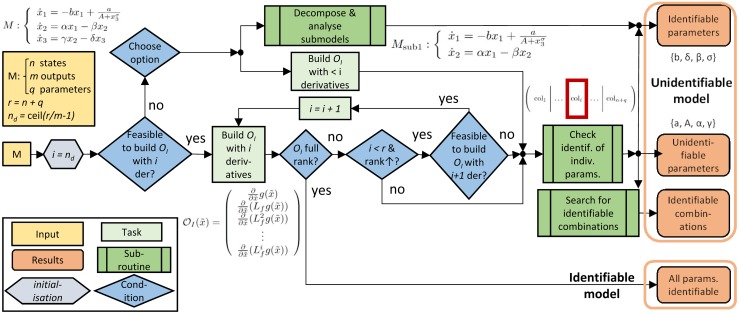
Core elements of the structural identifiability analysis method. Further refinements are possible: in some cases more complete solutions may be obtained by re-running the procedure after removing parameters already classified as identifiable. The model *M* used as example is the Goodwin oscillator analysed in the Results section.

Fig 1 shows a diagram of the methodology presented so far. The next sections discuss the types of analyses that can be performed with this methodology and show how to refine the solutions iteratively in order to obtain more complete diagnoses.

### Complete and partial analyses

By assessing identifiability as explained in sections “Assessing the OIC efficiently” and “Determining identifiability of individual parameters” we are performing a “Complete Case Analysis” (CCA): *every* parameter in the model is either classified as identifiable or as unidentifiable.

However, it may not always possible to carry out the aforementioned procedure due to computational limitations, as explained in sections “Decomposing large models into submodels to facilitate their analysis” and “Building $O_{I}$ with less than *n*_*d*_ Lie derivatives”, which presented two different alternatives. In certain cases these alternatives can yield incomplete results, that is, they may fail to determine the (un)identifiability of some parameters. For example, this may happen in the following scenarios:

1. When, due to computational limitations, $O_{I}$ is calculated with less Lie derivatives than those needed to guarantee identifiability or lack thereof. In this case, it may happen that an $O_{I}$ calculated with more Lie derivatives would have a higher rank, and therefore reveal the identifiability of more parameters.
2. When using decomposition, a parameter may not be determined as identifiable if the submodel in which it is being tested does not include some necessary states and outputs. Imagine, for example, that identification of a particular parameter *p*_*i*_ requires observing two outputs, *y*_*a*_ and *y*_*b*_, but only one of them was included in the submodel used to evaluate the identifiability of *p*_*i*_.

The two cases mentioned above will be called “Partial Analyses for Identifiability” (PAI): some parameters are conclusively classified as identifiable, but nothing can be said about the rest. It is also possible to perform similar analyses to guarantee unidentifiability of some parameters, leading to what we will call “Partial Analyses for Unidentifiability” (PAU). In such tests, some parameters are classified as unidentifiable while the analysis of the rest is not conclusive. This can happen in at least two situations:

1. Assume we are considering the full model with a full size matrix $O_{I}$ (i.e., a matrix built with as many Lie derivatives as states, *n*) or with a number of derivatives such that the rank of the matrix has stopped increasing. In this case, if we remove identifiable parameters we still have a CCA. However, if we remove parameters whose identifiability has not been assessed, the result of the subsequent rank test is conclusive only if it reports unidentifiability.
2. In the situation above, if instead of removing parameters we consider more outputs than are actually measured in the model.

The different types of analyses that can be performed are summarized in Table 1.

**Table 1:** Types of analyses possible with this methodology.

Analysis	# states	# derivatives, rank($O_{I}$)	other states	parameters	outputs
CCA	*s* = *n*	(*i* = *n*) OR (rank_i+1_ = rank_i_)			
PAI	*s* = *n*	(*i* < *n*) AND (rank_i_ > rank_i-1_)			
PAI	*s* < *n*		as unknown *p*		
PAU	*s* = *n*	(*i* = *n*) OR (rank_i+1_ = rank_i_)		removed s.u. *p*	
PAU	*s* = *n*	(*i* = *n*) OR (rank_i+1_ = rank_i_)			*o* > *m*

CCA: Complete Case Analysis; PAI: Partial Analysis for Identifiability; PAU: Partial Analysis for Unidentifiability; *i*: number of Lie derivatives used to build $O_{I}$; rank_*i*_: rank of $O_{I}$ with *i* derivatives; *s*: number of states taken into account; *n*: total number of states in the model; *o*: number of measured outputs; *m*: number of outputs in the original model; *x*: states; *p*: parameters. For detailed explanations, see section “Complete and partial analyses”. In all cases it is possible to remove from the model those parameters that have already been classified as identifiable, see section “Iterative refinement of the diagnosis”.

### Iterative refinement of the diagnosis

As shown in the preceding subsection, for some complex problems a complete analysis—that is, classifying all the parameters as identifiable or unidentifiable—may not be possible, at least in a first approach, due to computational limitations. In such cases, one can try to obtain more complete diagnoses by running the procedure iteratively. At each time, the computational cost can be reduced by removing from the augmented state vector, $\tilde{x}=[x,p]$, those parameters that were already found to be identifiable in previous steps. This operation, which leads to a smaller $O_{I}$ matrix, does not alter the result of the identifiability test, because the resulting $O_{I}$ is identical to the one obtained with the original vector $\tilde{x}=[x,p]$ after removing the columns corresponding to identifiable parameters—which results in a decreased rank. Note that this equivalence is made possible by the fact that $\dot{p}=0$, so the procedure cannot be applied to the model states, since $\dot{x}\neq 0$.

In summary, if a model M is too large to be analysed as a whole—i.e. to directly calculate the rank of its identifiability matrix and perform a complete case analysis (CCA)—identifiability analysis can be approached as follows:

1. Decompose the model, possibly (but not necessarily) using an optimization-based procedure to minimise computational effort, into several submodels, *S*_*i*_.
2. Analyse identifiability of the resulting *S*_*i*_ submodels using the generalised observability-identifiability rank condition. If the array is not full rank, test the identifiability of each parameter separately by comparing the rank before and after removing its column.
3. Parameters found to be identifiable in a submodel *S*_*i*_ are identifiable in the whole model M.
4. Several decompositions can be tested, which may lead to complementary results.
5. Additionally, as an alternative or a complement to steps (1–4), it may be possible to find identifiable parameters by checking the rank of a $O_{I}$ built with less than *n*_*d*_ Lie derivatives. Steps (1–5) correspond to what we call partial analyses for identifiability (PAI).
6. Remove all the parameters determined to be identifiable in the previous steps from the model M. This results in a reduction of the dimension of M which may enable its analysis using the generalised observability-identifiability rank condition without resorting to decomposition. In that case, it will be possible to determine the identifiability of all the parameters (CCA) or, alternatively, to perform a PAI.
7. For those parameters that are not classified as identifiable, try to assess their unidentifiability by performing the corresponding partial analysis, PAU.

### Implementation: The STRIKE-GOLDD toolbox

The present method has been implemented as a MATLAB toolbox named STRIKE-GOLDD (**STR**uctural **I**dentifiability ta**K**en as **E**xtended-**G**eneralized **O**bservability using **L**ie **D**erivatives and **D**ecomposition). It is an open source tool licensed under the GNU General Public License version 3 (GPLv3). It is freely available from https://sites.google.com/site/strikegolddtoolbox/ and as supplementary information accompanying this article (S1 File). It requires a MATLAB installation with the Symbolic toolbox. Additionally, to use optimization-based decomposition it is necessary to install the MEIGO toolbox [48]. The usage of the STRIKE-GOLDD software is discussed in detail in the manual (S2 Text); in the following lines we provide a brief description of the key options.

The toolbox allows limiting the number of Lie derivatives that are calculated when building $O_{I}\left(\tilde{x}\right)$. This is useful to prevent the algorithm from getting stuck in excessively lengthy calculations. To adapt this limit to the computer where the algorithm is running, it is specified as a machine-dependent criterion: the user can set a limit on the time invested in calculating these derivatives by entering it in opts.maxLietime (that is, the algorithm will not calculate the *i*^*th*^ + 1 derivative if the time spent in obtaining the *i*^*th*^ one was *t*_*i*_ > opts.maxLietime).

Furthermore, the user can choose what to do if this time limit is reached without $O_{I}\left(\tilde{x}\right)$ being full rank: by setting opts.decomp = 0, STRIKE-GOLDD will perform a partial analysis of the whole model with the current $O_{I}\left(\tilde{x}\right)$; if opts.decomp = 1, it will decompose the model. It is also possible to enforce the use of decomposition from the start, i.e. without checking whether the time limit is reached, with the option opts.forcedecomp. The submodels can be found by optimization or specified by the user; this choice is made by opts.decomp_user.

Another option, opts.numeric, allows deciding whether to calculate rank($O_{I}\left(\tilde{x}\right)$) numerically or symbolically. The symbolic calculation is always exact. It is possible to perform a numerical calculation by replacing the symbolic variables with prime numbers. This usually reduces the computational cost, although in some cases it might lead to accidental cancellations that decrease the rank artificially. However, the risk of obtaining a spurious result is low, and it can be minimized by running the procedure several times, since the substitutions are random. In all of the case studies analyzed in the Results section we found agreement between numeric and symbolic rank calculations.

Finally, it is possible to assess identifiability for specific values of the system’s initial conditions. As mentioned in subsection “Structural identifiability as augmented observability: the OIC”, this can be useful in order to detect situations in which loss of reachability from particular initial conditions leads to loss of identifiability. Such pathological cases are not detected if $\text{rank}(O_{I}\left({\tilde{x}}_{0}\right))$ is calculated using a generic symbolic vector of initial conditions. However, they can be tested by setting the option opts.knowninitc = 1 and entering the corresponding vector of initial conditions in the script that creates the model.

## Results

**Fig 2:**
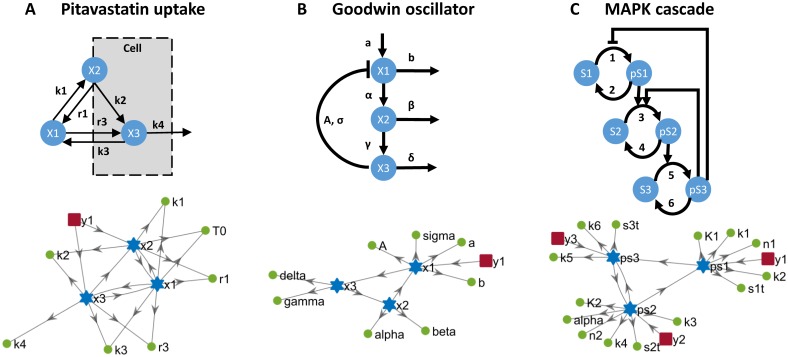
The first three models analysed in this work. (**A**) Pharmacokinetic model of Pitavastatin hepatic uptake. (**B**) Goodwin oscillator. (**C**) MAPK cascade with mixed feedback. The upper part of the figure shows functional diagrams of the three systems. The lower part shows the connections between the states (blue stars), outputs (red squares), and parameters (green circles); a directed arrow from X to Y indicates that Y appears in the dynamic equation of X.

**Fig 3:**
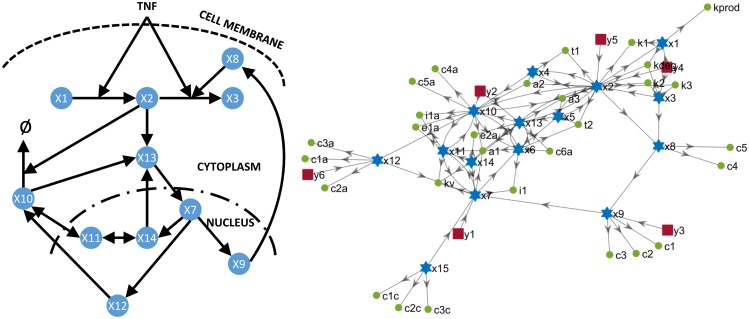
The NF-*κ*B model.

**Fig 4:**
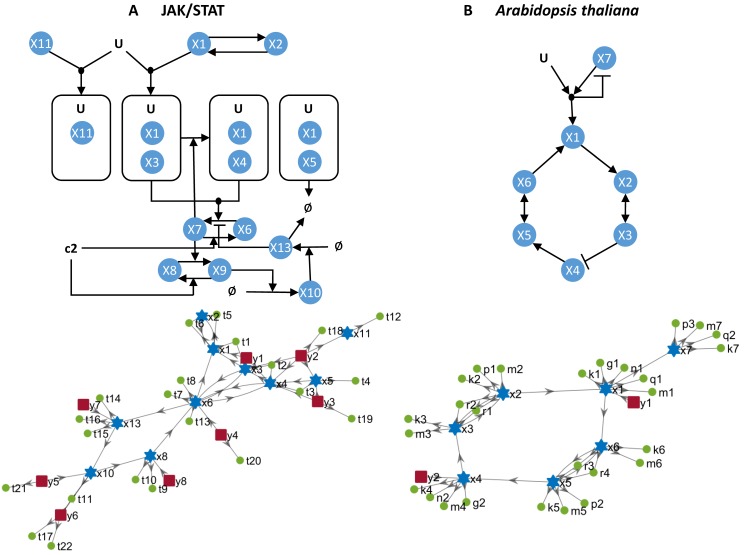
Models of JAK/STAT (A) and *Arabidopsis thaliana* (B). Note that states *x*_7_ and *x*_9_ in the upper diagram of JAK/STAT do not appear in the lower diagram since they are expressed as functions of *x*_6_ and *x*_8_ respectively.

We applied the proposed methodology to a number of published models of varying size and complexity [19, 27–32], some of which have been recently used to benchmark identifiability analysis methods [10, 19, 20]. The main features of the models are summarized in Table 2, and schematic diagrams are shown in Figs 2–4. Their equations are given in the supplementary information. Calculations were carried out on a computer running Windows7 SP1 64bit, with an Intel processor at 3.40 GHz and 16 GB of RAM, using MATLAB R2015b.

**Table 2:** Main features of the models analysed in this study.

Nr.	Description	Ref.	States	Outputs	Parameters	Identifiable
1	Pitavastatin hepatic uptake	[19]	3	1	7	yes
1.b	1 with steady state assumption	[19]	2	1	6	yes
2	Goodwin oscillator	[27]	3	1	8	no
3	MAPK with mixed feedback	[28]	3	3	14	yes
4	NF-*κ*B pathway	[30]	15	6	29	no
5	JAK/STAT pathway	[31]	10	8	23	no
6	Circadian clock *A. thaliana*	[29]	7	2	28	no
7	CCM of CHO cells	[32]	34	13	117	no

### Pharmacokinetic model of *in vitro* Pitavastatin hepatic uptake

In [19], Grandjean and coworkers proposed 18 alternative pharmacokinetic nonlinear compartmental models of the uptake process of Pitavastatin (a drug used to treat hypercholesterolaemia) into hepatocytes. They applied five different methods to analyse their structural identifiability: similarity transformation, differential algebra, Taylor series, and two approaches based on a non-differential input/output observable normal form and an algebraic input/output relationship approach. With these techniques they established the identifiability of most of the models. However, for several model formulations none of the methods was able to produce results. This was the case for two candidate models (with or without pseudo-state assumption) that accounted for drug metabolising within the cell.

A diagram of these Pitavastatin uptake models is shown in panel A of Fig 2. The upper part of the panel shows the system’s functional diagram. The lower part shows a graph drawn following the same convention as in [47], in which a directed arrow from A to B indicates that B appears in the dynamic equation of A. This graphical approach was originally proposed to study observability, and hence in [47] only the states were shown in the graphs. Since here we use it for identifiability purposes, we extend it to include both states and parameters (see figure caption for more details).

The method presented here determines that both Pitavastatin uptake models (with and without pseudo steady state assumption) are structurally identifiable.

### Enzymatic oscillations: The Goodwin model

The classical model of oscillations in enzyme kinetics proposed by Goodwin in 1965 [27] and shown in panel B of Fig 2 is still the subject of analyses [28]. It was selected by [10] to compare the performance of several structural identifiability methods, considering two different scenarios or variations of the model: when the three states are measured, or when only one of them—the enzyme concentration, *x*_1_—can be measured. The latter situation is more realistic, but its analysis is particularly challenging, and none of the methods tested by [10] managed to reach a conclusion due to computational complexity.

According to Eq (8), the minimum number of Lie derivatives for which the identifiability matrix may be full rank is *n*_*d*_ = 10 for this model. While the subsequent rank calculation is very demanding, the computational cost is substantially reduced by building $O_{I}\left(\tilde{x}\right)$ with only 9 Lie derivatives. In this way the method classifies four parameters as identifiable: *b*, *σ*, *β*, *δ*. Then, removing these parameters from the model as explained in “Iterative refinement of the diagnosis” enables the analysis of the remaining parameters (*a*, *A*, *α*, *γ*), which are found to be unidentifiable.

Thus this model is unidentifiable. It can be made identifiable by considering two parameters as known, one from each of the pairs {*A*, *a*} and {*α*, *γ*}. For example, if we fix the values of {*A*, *α*}, the remaining six unknown parameters in the model are identifiable. An alternative solution is to measure more states, if it is experimentally possible. In this case, if all three states are outputs, the model is structurally identifiable. Measuring only two of the three states, however, increases the number of identifiable parameters but does not render the model fully identifiable. The subsets of unidentifiable parameters for *y* = {*x*_1_, *x*_2_}, *y* = {*x*_1_, *x*_3_}, and *y* = {*x*_2_, *x*_3_} are, respectively, {*a*, *A*, *γ*}, {*α*, *γ*} and {*a*, *α*}.

### Three-layer MAPK cascade with mixed feedback

This model was presented in [28] as an example of a system exhibiting both oscillation and bistability. It is a three-layer signalling cascade with positive and negative feedback loops and Hill nonlinearities, shown in panel C of Fig 2. It has three states, which are the phosphorylated forms (*x*_1_, *x*_2_, *x*_3_), and 14 parameters (*k*_1_, *k*_2_, *k*_3_, *k*_4_, *k*_5_, *k*_6_, *s*_1*t*_, *s*_2*t*_, *s*_3*t*_, *K*_1_, *K*_2_, *n*_1_, *n*_2_, *α*).

This system requires that all its three states are measured in order to be identifiable. However, if just one of the states is left unmeasured, some parameters become unidentifiable: if *x*_1_ is not measured, *k*_3_ and *s*_1*t*_ are unidentifiable; if *x*_2_ is not measured, *k*_5_ and *s*_2*t*_ are unidentifiable; and if *x*_3_ is not measured, *K*_1_, *K*_2_, and *s*_3*t*_ are unidentifiable.

### NF-*κ*B signalling pathway

This model was presented by [30] and was used by both [10] and [16] as a benchmark for structural identifiability methods. In the formulation of [10], only 13 parameters are considered unknown. In that case, all of them are identifiable. The general case, in which all 29 parameters are in principle unknown, is more challenging. For this case STRIKE-GOLDD classifies 5 parameters as unidentifiable: *c*_1*c*_, *c*_2*c*_, *c*_3*c*_, *c*_4_, and *k*_2_, and the remaining as identifiable. Part of this diagnosis can be confirmed by inspection of the connection diagram in the right side of Fig 3, which shows that *c*_1*c*_, *c*_2*c*_, and *c*_3*c*_ only appear in the equation of state *x*_15_. Since *x*_15_ is in turn “disconnected” from the rest of the model (i.e. it does not appear in the equation of any other state), and it is not measured, there is clearly no way of determining its value. Hence *x*_15_ is unobservable, and the three parameters associated with it are unidentifiable. In contrast, the unidentifiability of *c*_4_ and *k*_2_ is by no means apparent from the figure. However, it can be determined with the methodology that they are not only unidentifiable, but related: fixing any of the two renders the other one identifiable. In summary, this 29-parameter model can be converted into a structurally identifiable 25-parameter model by fixing the values of four parameters: *c*_1*c*_, *c*_2*c*_, *c*_3*c*_, and either *c*_4_ or *k*_2_.

### JAK/STAT signalling pathway

This model of the IL13-Induced JAK/STAT signalling pathway was presented in [31] and later used in [20] to benchmark three identifiability analysis methods. The network interaction diagrams are shown in panel A of Fig 4. The results of our method coincide with those reported in [20], that is, five of the 23 parameters are unidentifiable, *p*_*u*_ = [*θ*_11_, *θ*_15_, *θ*_17_, *θ*_21_, *θ*_22_]. Following the procedure outlined in the Methods section, it is possible to find an identifiable combination of unidentifiable parameters. To do this we remove the columns corresponding to identifiable parameters and obtain a reduced submatrix, $O_{U}$. Calculation of a basis of the kernel of $O_{U}$ yields the following vector:

$$v=[0,0,\frac{-\theta_{17}}{\theta_{22}},0,1]$$ (11)

which in turn leads to the following PDE:

$$\frac{-\theta_{17}}{\theta_{22}}\cdot \frac{\partial \Phi }{\partial \theta_{17}}+\frac{\partial \Phi }{\partial \theta_{22}}=0\;\;\;\;\Rightarrow \;\;\;\;\Phi =\theta_{17}\cdot \theta_{22}$$ (12)

Thus, Φ = *θ*_17_ ⋅ *θ*_22_ is an identifiable parameter combination. The methodology does not report any combination involving *θ*_11_, *θ*_15_, *θ*_21_. If, additionally, we fix the value of *θ*_11_ *a priori*, we obtain a structurally identifiable model with 21 unknown parameters.

### Circadian clock in *Arabidopsis thaliana*

The genetic subnetwork that controls the circadian clock in the plant *A. thaliana* was modelled in [29]; its diagram is shown in Fig 4. This model uses both Michaelis-Menten and Hill kinetics. Two Hill coefficients of transcription (*a*, *b*) were considered as known parameters in the original publication [29]. Although it was argued in [29] that there is evidence that *b* = 2, coefficient *a* was fixed to *a* = 1 without experimental evidence. In [10] it was reported that (for the case of *a* = 1) no global structural identifiability method was capable of successfully analysing the model; at most, identifiability of five parameters was established.

While the choice of *a* = 1 makes the system rational and reduces the problem dimension, here we consider the more general case in which *a* is an unknown parameter. According to Eq (8), the minimum number of Lie derivatives for which $O_{I}\left(\tilde{x}\right)$ may be full rank is very high for this model (*n*_*d*_ = 16). This is the same situation as with the previously analysed Goodwin model, that is, the computational cost of the construction and subsequent rank calculation of $O_{I}\left(\tilde{x}\right)$ with *n*_*d*_ derivatives is too high. Furthermore, we found that the approach adopted for the Goodwin model—building the matrix with less than *n*_*d*_ derivatives—was not successful in the case of this example, at least when performed with few derivatives. Hence we turned to the alternative procedure, i.e. decomposing the model using optimization. In this way, identifiability of ten parameters was established: *a*, *k*_1_, *k*_4_, *m*_1_, *m*_4_, *n*_1_, *n*_2_, *q*_2_, *r*_2_, and *r*_4_. Removing these parameters from the model decreases the number of required derivatives *n*_*d*_ to 12, which is still very high; however, building $O_{I}\left(\tilde{x}\right)$ with 9 derivatives reports identifiability of an additional parameter, *r*_1_.

By performing partial analyses for unidentifiability (PAUs) we confirmed that the model is indeed unidentifiable. This can be remedied in several ways. A possible solution is to measure more states, if it is experimentally feasible. In the model it has been assumed that only mRNA concentrations are measured (i.e. states *x*_1_ and *x*_4_); however, if protein concentrations (i.e. the remaining states) are also measured, then all the parameters become structurally identifiable. Alternatively, if we assume that only the original outputs can be measured, it is possible to obtain an identifiable reformulation of the model by fixing some parameters. For example, choosing fixed values for the five degradation constants that were not found to be identifiable (*k*_2_, *k*_3_, *k*_5_, *k*_6_, *k*_7_) yields a structurally identifiable model with 23 parameters.

### Metabolic model of Chinese Hamster Ovary cell (CHO)

**Fig 5:**
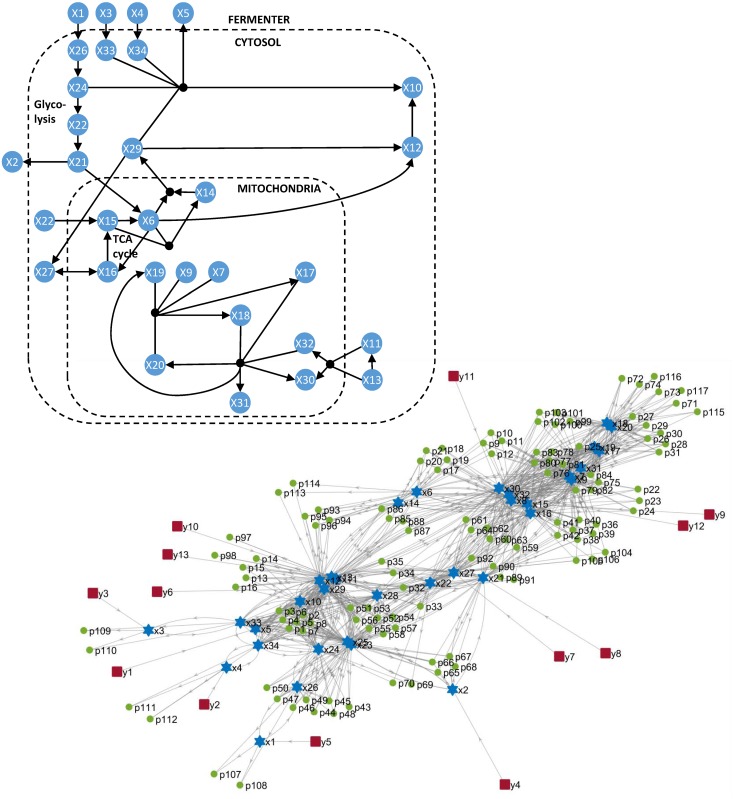
Metabolic model of Chinese Hamster Ovary cells used in a fed-batch fermentation process.

This large-scale model was taken from the BioPreDyn-bench collection [32], where it was included as benchmark B4. It models a batch fermentation process for protein production using Chinese Hamster Ovary cells. Its diagrams are shown in Fig 5. It contains 34 states (which are metabolites present in three compartments: fermenter, cytosol, and mitochondria), of which 13 are measured outputs. Its 32 reactions include protein product formation, the Embden-Meyerhof-Parnas pathway (EMP), the TCA cycle, a reduced amino acid metabolism, lactate production, and the electron transport chain. The reactions are modelled using lin-log kinetics [49], resulting in non-rational equations with 117 unknown parameters. While it was noted in [32] that the parameter estimation results suggested practical identifiability issues, possible deficiencies in structural identifiability were not mentioned. Given the size of this model, its analysis is very challenging.

Using decomposition it is possible to classify most of the parameters in the model as identifiable. However, we also found that at least four parameters are structurally unidentifiable: they are the parameters numbered 47, 48, 55, and 57, which correspond to the following kinetic constants (elasticities): {*e*_54_, − *e*_55_, − *e*_62_, *e*_64_}. After inspecting the model, we realised that it is possible to rewrite its dynamic equations in such a way that these parameters appear as (*e*_54_ + *e*_55_) and (*e*_62_ + *e*_64_); clearly, the individual parameters appearing in these sums are not identifiable. Thus, we replaced these four parameters with two new ones, *e*_*n*1_ = *e*_54_ + *e*_55_ and *e*_*n*2_ = *e*_62_ + *e*_64_. In this way we obtained a new model with 115 parameters, and confirmed that the newly introduced ones are structurally identifiable. Overall, we determined the identifiability of 97 parameters. While we did not manage to assess the identifiability of the remaining 18, we did find that fixing six of them (e.g. {*p*_28_, *p*_72_, *p*_77_, *p*_101_, *p*_105_, *p*_115_}) results in a structurally identifiable model. This action is slightly conservative, since those parameters can in principle be s.l.i. However, since the model has practical identifiability deficiencies [32] (as is typical of models of this type and size [49]), and given that it would be necessary to perform many Lie derivatives to relate these parameters to the model outputs, it is likely that in practice their values will be difficult to estimate. Therefore, fixing a subset of them appears as a reasonable solution.

In summary, we found that: (i) this model is structurally unidentifiable, (ii) there exist two identifiable combinations of parameters, which convert 4 unidentifiable parameters into 2 identifiable ones, (iii) of the remaining 113 parameters, at least 95 are identifiable, and (iv) fixing the values of 6 parameters ensures that the remaining 12 (and the model as a whole) are identifiable.

## Discussion

We have presented a methodology for analysing the structural identifiability of dynamic models described by a system of ordinary differential equations. It builds on concepts and techniques originally presented in the context of nonlinear observability analysis. More specifically, it adopts a differential geometry approach, which is based on building an augmented observability matrix—with the parameters considered as additional state variables—and calculating its rank. This formulation, as opposed to other approaches based on differential algebra, allows handling any analytic models, without requiring them to be in rational or polynomial form. If a model is structurally unidentifiable the method determines the identifiability of each parameter individually, by recalculating the matrix rank after removing the corresponding column.

Realising that the structural identifiability analysis of nonlinear dynamic models is a challenging task, and that this difficulty increases rapidly with the problem size, our method is geared towards computational efficiency. To this end it includes several algorithmic developments to facilitate the analysis of models of larger size. One is the possibility of decomposing the model into smaller submodels, which can be found by optimization or specified by the user. Another is the calculation of the matrix rank with a reduced number of Lie derivatives. These alternatives lead in some cases to partial analyses, whose result is only conclusive if a parameter is classified as identifiable, but not as unidentifiable (or vice versa, depending on the type of analysis). In these situations the method also allows for an iterative refinement of the diagnosis: by removing parameters already classified as identifiable, the problem size is reduced and more complete analyses are made possible.

To facilitate the application of this methodology, we have provided it as a free MATLAB (The MathWorks, Natick, MA) toolbox called STRIKE-GOLDD (STRuctural Identifiability taKen as Extended-Generalized Observability with Lie Derivatives and Decomposition), available under the GNU General Public License from https://sites.google.com/site/strikegolddtoolbox/. We expect that STRIKE-GOLDD will contribute to fill the gap between the complexity of current systems biology models and their usability, which can be compromised unless structural identifiability is assessed.

We have validated the methodology using a set of nonlinear systems biology models whose size and/or complexity make them challenging case studies. They range from a classic model of enzymatic oscillations with 8 parameters proposed by Goodwin in 1965 [27] to a metabolic model of more than 100 parameters published in 2015 [32]. Interestingly, we found structural identifiability issues even in models of relatively small size, such as the aforementioned Goodwin model. Indeed, the results show that identifiability issues are likely to appear in over-parameterized models (with many parameters per state), specially if only few of their states are available for measurement (in order words, if there are few outputs).

A large parameter-to-output ratio also implies that the structural identifiability of the model will be difficult to analyse, because it will be necessary to perform many Lie derivative calculations in order to build the augmented observability matrix, thus incurring a high computational cost. Could this common cause mean that the difficulty in analysing a model is a hint of lack of identifiability? We ask this question because we know that, on the other hand, it is possible to analyse models with many parameters as long as sufficient measurements are available.

Among the models analysed here, the JAK/STAT pathway had already been studied [20], and for that case our method confirmed previously reported results. In other cases we established the identifiability of systems that had not been analysed before, such as the mixed feedback MAPK pathway [28] or the model of Pitavastatin hepatic uptake (which had been reported to resist analysis when attempted with other methods, although it was suspected that it was identifiable [19]). Perhaps more interestingly, we also found some unidentifiabilities that had not been previously reported. An example is the Goodwin oscillator [27], for which it was established that half of its parameters are structurally unidentifiable. Despite the relatively small size of this model (3 states and 8 parameters), the fact that it is not a rational system, combined with the high parameter-to-output ratio (given that only one of its states is measured) make it a very challenging problem. Similar issues were found in the NF-*κ*B signalling pathway [30] and in the genetic subnetwork of the circadian clock in *Arabidopsis thaliana* [29]. In these cases it can be noted that the ratio of unidentifiable parameters is larger in models with a lower ratio of measured outputs. Finally, we also detected unidentifiabilities in a recently presented large-scale dynamic model of metabolism of Chinese Hamster Ovary cells [32] with 117 parameters.

We have also shown how to turn unidentifiable models into identifiable ones. With the procedure described in this paper it is sometimes possible to combine several unidentifiable parameters into a single identifiable combination. More often the solution is to reparameterize the model by considering some of the unidentifiable parameters as known constants, fixing them to values that appear reasonable according to available knowledge. In this way the remaining unknown parameters are rendered identifiable. Finally, a model can also be made identifiable by increasing the number of its outputs, if it is experimentally possible to measure more of its states.

## Supporting Information

S1 Text Mathematical details of the models used as case studies. (PDF)

S2 Text STRIKE-GOLDD documentation. User manual of the toolbox.
(PDF)

S1 File STRIKE-GOLDD software. The MATLAB toolbox implementing the methodology. It is also available at https://sites.google.com/site/strikegolddtoolbox/.
(ZIP)
