# Supplementary material for: Structural Identifiability of Dynamic Systems Biology Models
Source: PLoS Comput Biol. 2016 Oct 28;12(10):e1005153. doi: 10.1371/journal.pcbi.1005153 (PMC5085250; doi:10.1371/journal.pcbi.1005153)
Supplement: S1 Text — (PDF) [file pcbi.1005153.s001.pdf]

# Structural identifiability of dynamic systems biology models: Supplementary Information File S1

Alejandro F. Villaverde[1,2 *], Antonio Barreiro[2], and Antonis Papachristodoulou[1]

[1]Department of Engineering Science, University of Oxford, Oxford OX1 3PJ, UK
[2]Department of Systems & Control Engineering, University of Vigo, Vigo 36310, Spain
[*]e-mail: afernandez@uvigo.es

June 2, 2016

## 1 Mathematical details of the models used as case studies.

### 1.1 Pharmacokinetic model of *in vitro* Pitavastatin hepatic uptake

This pharmacokinetic nonlinear compartmental model of the uptake process of Pitavastatin (a drug used to treat hypercholesterolaemia) into hepatocytes was proposed in [1]. This is one of the two candidate models that accounted for drug metabolism within the cell. It has 3 states, 1 output ($y = k(x_2 + x_3)$), and 7 unknown parameters ($k_1, k_2, k_3, k_4, r_1, r_3, T_0$):

$$\left\{ \begin{array}{l} \dot{x}_1 = k_3 x_3 - r_3 x_1 - k_1 x_1 (T_0 - x_2) + r_1 x_2, \\ \dot{x}_2 = k_1 x_1 (T_0 - x_2) - (r_1 + k_2) x_2, \\ \dot{x}_3 = r_3 x_1 - (k_3 + k_4) x_3 + k_2 x_2, \\ x_1(0) = D,\ x_2(0) = 0,\ x_3(0) = 0 \end{array} \right. \tag{1}$$

The constants $k, D$ are assumed known.

A different version of this model was proposed making a pseudo steady state assumption. In this case the number of states reduces to two:

$$\left\{ \begin{array}{l} \dot{x}_1 = k_3 x_3 - r_3 x_1 - \frac{V_M x_1}{K_M + x_1}, \\ \dot{x}_3 = r_3 x_1 - (k_3 + k_4) x_3 + \frac{V_M x_1}{K_M + x_1}, \\ x_1(0) = D_1,\ x_3(0) = 0 \end{array} \right. \tag{2}$$

with an output $y = k(\frac{T_0 x_1}{K_M + x_1} + x_3)$, and 6 unknown parameters ($V_M, K_M, k_3, k_4, r_3, T_0$). Similarly, $D_1$ and $k$ are known constants.

### 1.2 Enzymatic oscillations: the Goodwin model

This classic model of oscillations in enzyme kinetics was proposed by [2] and used by [3] to benchmark several global structural identifiability methods. The model has 1 output ($y = x_1$), 3 states, and 8 parameters:

$$\left\{ \begin{array}{l} \dot{x}_1 = -b x_1 + \frac{a}{A + x_3^{\sigma}} \\ \dot{x}_2 = \alpha x_1 - \beta x_2 \\ \dot{x}_3 = \gamma x_2 - \delta x_3 \\ x_1(0) = 0.3617,\ x_2(0) = 0.9137,\ x_3(0) = 1.3934 \end{array} \right. \tag{3}$$

### 1.3 Three-layer MAPK cascade with mixed feedback

This model was presented in [4] as an example of a system exhibiting both oscillation and bistability. It is a three-layer signalling cascade with positive and negative feedback loops and Hill nonlinearities. It has three states, which are the phosphorylated forms $(x_1, x_2, x_3)$, and 14 parameters ($k_1$, $k_2$, $k_3$, $k_4$, $k_5$, $k_6$, $s_{1t}$, $s_{2t}$, $s_{3t}$, $K_1$, $K_2$, $n_1$, $n_2$, $\alpha$):

$$\begin{cases} \dot{x}_1 = k_1(S_{1t} - x_1)(\frac{K_1^{n_1}}{K_1^{n_1}+x_3^{n_1}}) - k_2 x_1, \\ \dot{x}_2 = k_3(S_{2t} - x_2)x_1(1 + \frac{\alpha x_3^{n_2}}{K_2^{n_2}+x_3^{n_2}}) - k_4 x_2, \\ \dot{x}_3 = k_5(S_{3t} - x_3)x_2 - k_6 x_3, \end{cases} \tag{4}$$

### 1.4 NF-$\kappa$B signalling pathway

This model was presented by [5]. It has 15 states, 6 outputs, and 29 parameters:

$$\begin{cases} \dot{x}_1 = k_{prod} - k_{deg}x_1 - k_1 x_1 u(t), \\ \dot{x}_2 = -k_3 x_2 - k_{deg}x_2 - a_2 x_2 x_{10} + t_1 x_4 - a_3 x_2 x_{13} + t_2 x_5 + (k_1 x_1 - k_2 x_2 x_8)u(t), \\ \dot{x}_3 = k_3 x_2 - k_{deg}x_3 + k_2 x_2 x_8 u(t), \\ \dot{x}_4 = a_2 x_2 x_{10} - t_1 x_4, \\ \dot{x}_5 = a_3 x_2 x_{13} - t_2 x_5, \\ \dot{x}_6 = c_{6a} x_{13} - a_1 x_6 x_{10} + t_2 x_5 - i_1 x_6, \\ \dot{x}_7 = i_1 kv x_6 - a_1 x_{11} x_7, \\ \dot{x}_8 = c_4 x_9 - c_5 x_8, \\ \dot{x}_9 = c_2 + c_1 x_7 - c_3 x_9, \\ \dot{x}_9 = c_2 + c_1 x_7 - c_3 x_9, \\ \dot{x}_{10} = -a_2 x_2 x_{10} - a_1 x_{10} x_6 + c_{4a} x_{12} - c_{5a} x_{10} - i_{1a} x_{10} + e_{1a} x_{11}, \\ \dot{x}_{11} = -a_1 x_{11} x_7 + i_{1a} kv x_{10} - e_{1a} kv x_{11}, \\ \dot{x}_{12} = c_{2a} + c_{1a} x_7 - c_{3a} x_{12}, \\ \dot{x}_{13} = a_1 x_{10} x_6 - c_{6a} x_{13} - a_3 x_2 x_{13} + e_{2a} x_{14}, \\ \dot{x}_{14} = a_1 x_{11} x_7 - e_{2a} kv x_{14}, \\ \dot{x}_{15} = c_{2c} + c_{1c} x_7 - c_{3c} x_{15}. \end{cases} \tag{5}$$

The outputs are: $y_1 = x_7$, $y_2 = x_{10} + x_{13}$, $y_3 = x_9$, $y_4 = x_1 + x_2 + x_3$, $y_5 = x_2$, $y_6 = x_{12}$. It was used both by [3] and [6] as a benchmark for structural identifiability methods. In the formulation of [3], only 13 parameters are considered unknown. In general, all of them can be considered unknown.

### 1.5 JAK/STAT signalling pathway

This model of the IL13-Induced JAK/STAT signalling pathway was presented by [7] and later used by [8]:

$$\begin{cases} \dot{x}_1 = \theta_6 x_2 - \theta_5 x_1 - \frac{453\,\theta_1\,u_1\,x_1}{200}, \\ \dot{x}_2 = \theta_5 x_1 - \theta_6 x_2, \\ \dot{x}_3 = \theta_2 x_3 \left(x_6 - \frac{14}{5}\right) + \frac{453\,\theta_1\,u_1\,x_1}{200}, \\ \dot{x}_4 = -\theta_3 x_4 - \theta_2 x_3 \left(x_6 - \frac{14}{5}\right), \\ \dot{x}_5 = \theta_3 x_4 - \theta_4 x_5, \\ \dot{x}_6 = -91\,\theta_8 \left(x_6 - \frac{14}{5}\right) - \frac{\theta_7\,x_3\,x_6}{\theta_{13}\,x_1+1} - \frac{\theta_7\,x_4\,x_6}{\theta_{13}\,x_{13}+1}, \\ \dot{x}_8 = \theta_9 x_8 \left(x_6 - \frac{14}{5}\right) - 91\,\theta_{10} \left(x_8 - 165\right), \\ \dot{x}_{10} = -\theta_{11} \left(x_8 - 165\right), \\ \dot{x}_{11} = -\frac{453\,\theta_{12}\,u_1\,x_{11}}{200}, \\ \dot{x}_{13} = \frac{\theta_{14}\,x_{10}}{\theta_{15}+x_{10}} - \theta_{16}\,x_{13}, \\ \mathbf{x}(0) = [1.3, \theta_{23}, 0, 0, 0, 2.8, 165, 0, 0.34, 0] \end{cases} \tag{6}$$

The outputs are: $y_1 = x_1+x_3+x_4$, $y_2 = \theta_{18}(x_3+x_4+x_5+0.34-x_{11})$, $y_3 = \theta_{19}(x_4+x_5)$, $y_4 = \theta_{20}(2.8-x_6)$, $y_5 = \theta_{21}x_{10}$, $y_6 = \theta_{17}\theta_{22}x_{10}/\theta_{11}$, $y_7 = x_{13}$, and $y_8 = 165 - x_8$.

## 1.6 Circadian clock in *Arabidopsis thaliana*

The genetic network that controls the circadian clock in *A. thaliana* was proposed in [9]. This model has 2 outputs ($y_1 = x_1, y_2 = x_4$), 7 states, and 28 parameters:

$$\begin{cases} \dot{x}_1 = n_1 \frac{x_6^a}{g_1^a + x_6^a} - m_1 \frac{x_1}{k_1 + x_1} + q_1 x_7 u(t), \\ \dot{x}_2 = p_1 x_1 - r_1 x_2 + r_2 x_3 - m_2 \frac{x_2}{k_2 + x_2}, \\ \dot{x}_3 = r_1 x_2 - r_2 x_3 - m_3 \frac{x_3}{k_3 + x_3}, \\ \dot{x}_4 = n_2 \frac{g_2^2}{g_2^2 + x_3^2} - m_4 \frac{x_4}{k_4 + x_4}, \\ \dot{x}_5 = p_2 x_4 - r_3 x_5 + r_4 x_6 - m_5 \frac{x_5}{k_5 + x_5}, \\ \dot{x}_6 = r_3 x_5 - r_4 x_6 - m_6 \frac{x_6}{k_6 + x_6}, \\ \dot{x}_7 = p_3 - m_7 \frac{x_7}{k_7 + x_7} - (p_3 + q_2 x_7) u(t) \\ x_i(0) = 0; i = 1, \ldots, 7. \end{cases} \tag{7}$$

## 1.7 Metabolic model of Chinese Hamster Ovary cell (CHO)

This metabolic model of Chinese Hamster Ovary was included in the BioPreDyn-bench collection [10] as benchmark B4. It has 34 states, of which 13 are outputs, and 117 parameters. Due to its large size the equations are not included here; the reader is referred to the supplementary information of the original publication [10] for details (it can be freely accessed online).

# References


[1] Grandjean TR, Chappell MJ, Yates JW, Evans ND. Structural identifiability analyses of candidate models for in vitro Pitavastatin hepatic uptake. Comput Methods Programs Biomed. 2014;114(3):e60–e69.

[2] Goodwin BC. Oscillatory behavior in enzymatic control processes. Adv Enzyme Regul. 1965;3:425–437.

[3] Chiş OT, Banga JR, Balsa-Canto E. Structural identifiability of systems biology models: a critical comparison of methods. PLoS One. 2011;6(11):e27755.

[4] Nguyen LK, Degasperi A, Cotter P, Kholodenko BN. DYVIPAC: an integrated analysis and visualisation framework to probe multi-dimensional biological networks. Sci Rep. 2015;5.

[5] Lipniacki T, Paszek P, Brasier AR, Luxon B, Kimmel M. Mathematical model of NF-$\kappa$B regulatory module. J Theor Biol. 2004;228(2):195–215.

[6] Karlsson J, Anguelova M, Jirstrand M. An Efficient Method for Structural Identiability Analysis of Large Dynamic Systems. In: 16th IFAC Symposium on System Identification. vol. 16; 2012. p. 941–946.

[7] Raia V, Schilling M, Böhm M, Hahn B, Kowarsch A, Raue A, et al. Dynamic mathematical modeling of IL13-induced signaling in Hodgkin and primary mediastinal B-cell lymphoma allows prediction of therapeutic targets. Cancer Res. 2011;71(3):693–704.

[8] Raue A, Karlsson J, Saccomani MP, Jirstrand M, Timmer J. Comparison of approaches for parameter identifiability analysis of biological systems. Bioinformatics. 2014;p. btt006.

[9] Locke J, Millar A, Turner M. Modelling genetic networks with noisy and varied experimental data: the circadian clock in Arabidopsis thaliana. J Theor Biol. 2005;234(3):383–393.

[10] Villaverde AF, Henriques D, Smallbone K, Bongard S, Schmid J, Cicin-Sain D, et al. BioPreDyn-bench: a suite of benchmark problems for dynamic modelling in systems biology. BMC Syst Biol. 2015;9(1):8.
