# Supplementary material for: Structural Identifiability of Dynamic Systems Biology Models
Source: PLoS Comput Biol. 2016 Oct 28;12(10):e1005153. doi: 10.1371/journal.pcbi.1005153 (PMC5085250; doi:10.1371/journal.pcbi.1005153)
Supplement: S2 Text — User manual of the toolbox. (PDF) [file pcbi.1005153.s002.pdf]

STRIKE-GOLDD USER MANUAL

STRuctural Identifiability taKen as Extended-Generalized Observability with Lie Derivatives and Decomposition

Alejandro F. Villaverde[1,2] *

[1]Department of Engineering Science, University of Oxford
[2]Department of Systems & Control Engineering, Universidade de Vigo
[*]e-mail: afernandez@uvigo.es

May 18, 2016

With the collaboration of:
Antonio Barreiro[2]
Antonis Papachristodoulou[1]

Contents

1 Introduction 1

2 License 1

3 Availability 1

4 Software contents 1

5 Requirements and installation 2
- 5.1 Requirements 2
- 5.2 Download and install 2

6 Quick start: using STRIKE-GOLDD in one minute 3

7 Usage 3
- 7.1 Input: entering a model 3
- 7.2 Options 4
- 7.3 Output 5

8 Acknowledgements 6

9 References 6

1 Introduction

STRIKE-GOLDD is a MATLAB toolbox that analyses the local structural identifiability of a (possibly nonlinear) dynamic model.

It follows a differential geometry approach, recasting the identifiability problem as an observability problem. Essentially, identifiability is determined by calculating the rank of a generalized observability-identifiability matrix, which is built using Lie derivatives. When this rank test classifies a model as unidentifiable, the procedure determines the subset of identifiable parameters. In some cases it is also possible to find identifiable combinations of the remaining parameters.

This approach is directly applicable to many models of small and medium size; larger systems can be analysed using additional features of the method. One of them is decomposition into more tractable submodels, which is performed with a combinatorial optimization metaheuristic. Another possibility is to build identifiability matrices with a reduced number of Lie derivatives.

Parts of the methodology implemented in STRIKE-GOLDD were presented in [1]. Optimization is performed with the Variable Neighbourhood Search (VNS) metaheuristic [2] included in the MEIGO toolbox [3].

2 License

STRIKE-GOLDD is licensed under the GNU General Public License version 3 (GPLv3), a free, copyleft license for software.

3 Availability

STRIKE-GOLDD can be downloaded from:
`https://sites.google.com/site/strikegolddtoolbox/`.

4 Software contents

The STRIKE-GOLDD toolbox consists of several MATLAB files, organized as follows:

Root folder (`/STRIKE-GOLDD`):

- `STRIKE-GOLDD.m` is the main file. Running it will execute STRIKE-GOLDD.
- `options.m` is the file that the user must edit in order to specify the problem to solve and the options for solving it.

Functions folder (`/STRIKE-GOLDD/functions`):

- `combin_optim.m`: performs combinatorial optimization using the Variable Neighbourhood Search metaheuristic (VNS) [2] from the MEIGO toolbox [3].

- `combos.m`: finds identifiable combinations of otherwise unidentifiable parameters.
- `decomp.m`: decomposes the model into submodels (either defined by the user, or found via optimization) and analyses them.
- `elim_and_recalc.m`: determines identifiability of individual parameters one by one, by successive elimination of its column in the identifiability matrix and recalculation of its rank.
- `graph_model.m`: creates a graph showing the relations among model states, outputs, and parameters.
- `objective_fun.m`: calculates the objective function value in the optimization (as the ratio between number of model outputs and parameters, plus a penalty on the number of states).
- `primes10k.mat`: MATLAB data file that stores 10000 prime numbers, used in numerical rank calculations.

Two additional folders, `/STRIKE-GOLDD/models` and `/STRIKE-GOLDD/results`, store the input and output files respectively.

5 Requirements and installation

5.1 Requirements

STRIKE-GOLDD can run on any operating system compatible with MATLAB.
Apart from a MATLAB installation, the additional requisites are:

- The MATLAB Symbolic Math Toolbox.
- The MATLAB MEIGO toolbox [3], which can be freely downloaded from `http://gingproc.iim.csic.es/meigom.html`. The MEIGO toolbox is only needed if the optimization-based model decomposition is used.

It has been tested with MATLAB versions R2014b and R2015b.

5.2 Download and install

1. Download STRIKE-GOLDD from:
   `https://sites.google.com/site/strikegolddtoolbox/`.
2. Unzip the STRIKE-GOLDD folder.
3. Download the MEIGO toolbox from: `http://gingproc.iim.csic.es/meigom.html`.
4. Unzip the MEIGO folder.
5. Tell STRIKE-GOLDD about the location of MEIGO by modifying the corresponding line in the `options.m` file as follows (you must replace the example below with the actual location in your computer):
   `paths.meigo = 'C:\Users\My_name\Documents\MEIGO_M-v03-07-2014\MEIGO_M';`

6 Quick start: using STRIKE-GOLDD in one minute

To start using STRIKE-GOLDD you only need to follow these steps:

1. Follow the installation instructions in Section 5.2.
2. Open a MATLAB session and go to the STRIKE-GOLDD root directory ("STRIKE-GOLDD").
3. Define the problem and options by editing the script `options.m` (see Section 7.1 for details).
   - QUICK DEMO EXAMPLE: If you are running STRIKE-GOLDD for the first time and/or just want to see how it works, you can skip this step and leave `options.m` unedited. This will analyse a model of the JAK/STAT signalling pathway with default options.
4. Run `STRIKE-GOLDD.m` (to do this you can either type "STRIKE-GOLDD" in the command window, or right-click `STRIKE-GOLDD.m` in the "Current Directory" tab and select "run").

Done! Results will be reported in the MATLAB screen. A screenshot of an execution is shown in Figure 1.

7 Usage

7.1 Input: entering a model

STRIKE-GOLDD reads models stored as MATLAB MAT-files (.mat). The model states, outputs, and parameters, as well as its dynamic equations, must be defined as vectors of symbolic variables, whose names must follow a specific convention.

Here we show how to do it, using the MAPK model included in the `models` folder as an example. The file read by STRIKE-GOLDD is `MAPK.mat`. This file, which stores the model variables, can be created from the M-file `z_create_MAPK_model.m`. In the following lines we comment the different parts of the M-file, illustrating the process of defining a suitable model.

First, all the parameters, states, and any other entities (such as inputs or known constants) appearing in the model must be defined as symbolic variables:

```
syms k1 k2 k3 k4 k5 k6 ...
     ps1 ps2 ps3 ...
     s1t s2t s3t ...
     KK1 KK2 n1 n2 alpha  ...
```

Then we define the state variables, by creating a column vector named x:

```
x    = [ps1; ps2; ps3];
```

Similarly, we define the vector of output variables, which must be named h (in this case they coincide with the state variables):

```
h     = x;
```

The vector of unknown parameters must be called p:

```
p     = [k1; k2; k3; k4; k5; k6;s1t; s2t; s3t; KK1; KK2; n1; n2; alpha];
```

The dynamic equations must also be entered as a column vector, called f:

```
f     = [k1*(s1t-ps1)*(KK1^n1)/(KK1^n1+ps3^n1)-k2*ps1;
           k3*(s2t-ps2)*ps1*(1+(alpha*ps3^n2)/(KK2^n2+ps3^n2))-k4*ps2 ;
           k5*(s3t-ps3)*ps2-k6*ps3 ];
```

The vector of initial conditions must be called *ics*. If they are unknown, enter a blank vector:

```
ics  = [ ];
```

Additionally we define another vector, `known_ics`, to specify which initial conditions are known. It must have the same length as the state vector x, and its entries should be either 1 or 0, depending on whether the corresponding initial condition is known or unknown, respectively:

```
known_ics = [0,0,0];
```

Finally, save all the variables in a MAT-file:

```
save('MAPK','x','h','p','f','ics','known_ics');
```

7.2 Options

The model to analyse, as well as the options for performing the analysis, are entered in the `options.m` file. All the options are set to default values which can be modified by the user or left unchanged. In the `options.m` file the options are classified in five blocks as follows:

(1) The first block consists of solely one option, the name of the model to analyse. By default it is set to one of the nine models provided with the toolbox, the JAK/STAT signalling pathway:

```
model = 'JAKSTAT';
```

The user may select other models provided with the toolbox – included in folder `models` – or define a new model as explained in Section 7.1.

(2) The second block specifies some paths. The user only needs to modify one of them, the path of the MEIGO toolbox (although even this can be skipped if the model is *not* going to be

decomposed using optimization):

```
paths.meigo  = 'C:\Users\My_name\Documents\MEIGO_M-v03-07-2014\MEIGO_M';
```

(3) The third block consists of the following options: `opts.numeric`, `opts.knowninitc`, `opts.initCond`, `opts.findcombos`, `opts.unidentif`, `opts.forcedecomp`, `opts.decomp`, `opts.decomp_user`, `opts.maxLietime`, `opts.maxOpttime`, `opts.maxstates`, which can be modified or left to their default values. Their meaning is explained in the comments of the `options.m` file.

(4) The fourth block defines submodels to analyse. They should only be specified in this way if the user wants to define them manually instead of relying on the optimisation algorithm. In the former case, every submodel must be specified as a vector containing the indices of the states included in it. For example, the following lines define two submodels, consisting of states [x(1), x(2)] and [x(2), x(3)], respectively:

```
submodels    = [];
submodels{1} = [1 2];
submodels{2} = [2 3];
```

(5) The fifth block is used for entering parameters that have already been classified as identifiable. This reduces the computational complexity of the calculations and may thus enable a deeper analysis, which can lead to more complete results. For example, if STRIKE-GOLDD has already determined that two parameters p_1 and p_2 are identifiable, we may enter:

```
syms p1 p2
prev_ident_pars = [p1 p2];
```

7.3 Output

STRIKE-GOLDD reports the main results of the identifiability analysis on screen.

Additionally, it creates several MAT-files in the `results` folder:

- A file named `id_results_MODELNAME_DATE.mat`, with the results of the identifiability analysis and most of the intermediate results. The main results are: `p_id` (list of identifiable parameters), `p_un` (unidentifiable parameters), `obs_states` (observable states), and `unobs_states` (unobservable states).
- One or several files named `obs_ident_matrix_MODEL_NUMBER_OF_Lie_deriv.mat`, with the generalized observability-identifiability matrices calculated with a given number of Lie derivatives. They are stored in separate files so that they can be reused in case a particular run is aborted due to excessive computation time.
- If decomposition is used, STRIKE-GOLDD creates a subfolder in the `results` folder named `decomp_MODEL_DATE_MAXSTATES_MAXLIETIME` (i.e., it specifies the model name, the date,

the maximum number of states allowed in every submodel, and the maximum time allowed for performing a Lie derivative). Inside the folder it stores one MAT-file per submodel with partial results. Additionally, the same MAT-file as described in the previous point is created in the `results` folder.

8 Acknowledgements

STRIKE-GOLDD has received funding from the Galician government (Xunta de Galiza) through the I2C postdoctoral program, fellowship ED481B2014/133-0, and by the Spanish Ministry of Economy and Competitiveness (MINECO), grant DPI2013-47100-C2-2-P.

9 References

[1] Villaverde AF, Barreiro A, Papachristodoulou A. Structural Identifiability Analysis via Extended Observability and Decomposition. In: 6th IFAC Conference on Foundations of Systems Biology in Engineering; 2016. Submitted.

[2] Mladenović N, Hansen P. Variable neighborhood search. Comput Oper Res. 1997;24(11):1097–1100.

[3] Egea J, Henriques D, Cokelaer T, Villaverde AF, MacNamara A, Danciu DP, et al. MEIGO: an open-source software suite based on metaheuristics for global optimization in systems biology and bioinformatics. BMC Bioinf. 2014;15:136.

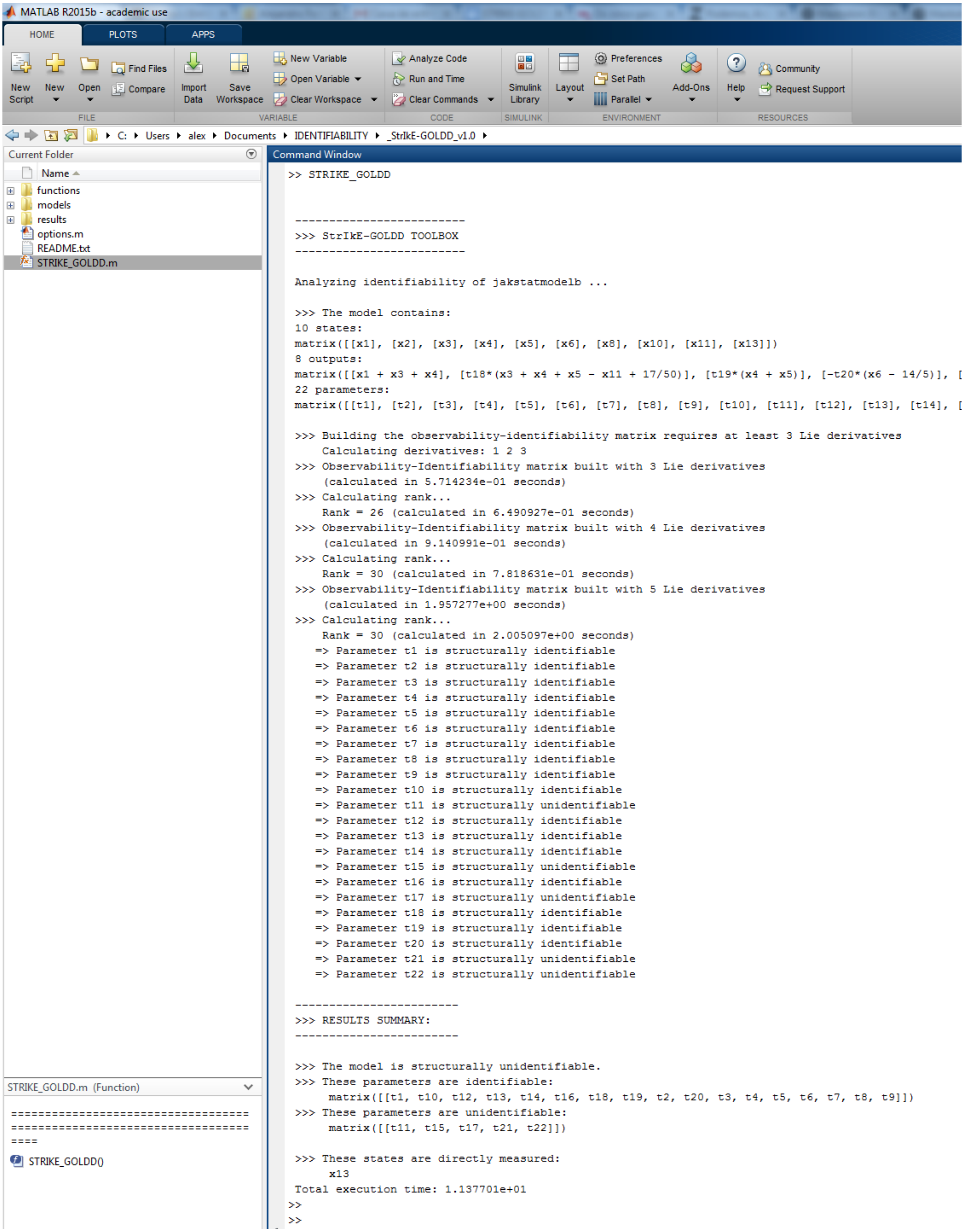

Figure 1: **Screen shot of a STRIKE-GOLDD run.**
